# The Role of Exercise-Induced Molecular Processes and Vitamin D in Improving Cardiorespiratory Fitness and Cardiac Rehabilitation in Patients With Heart Failure

**DOI:** 10.3389/fphys.2021.794641

**Published:** 2022-01-11

**Authors:** Aneta Aleksova, Milijana Janjusevic, Giulia Gagno, Alessandro Pierri, Laura Padoan, Alessandra Lucia Fluca, Cosimo Carriere, Antonio Paolo Beltrami, Gianfranco Sinagra

**Affiliations:** ^1^Cardiothoracovascular Department, Azienda Sanitaria Universitaria Giuliano Isontina (ASUGI), University of Trieste, Trieste, Italy; ^2^Department of Medical Surgical and Health Science, University of Trieste, Trieste, Italy; ^3^Cardiology and Cardiovascular Physiopathology, Azienda Ospedaliero-Universitaria S. Maria della Misericordia, Perugia, Italy; ^4^Department of Clinical Pathology, Azienda Sanitaria Universitaria Friuli Centrale (ASUFC), University of Udine, Udine, Italy; ^5^Department of Medicine (DAME), University of Udine, Udine, Italy

**Keywords:** exercise, heart failure, hypertrophy, inflammation, vitamin D

## Abstract

Heart failure (HF) still affects millions of people worldwide despite great advances in therapeutic approaches in the cardiovascular field. Remarkably, unlike pathological hypertrophy, exercise leads to beneficial cardiac hypertrophy characterized by normal or enhanced contractile function. Exercise-based cardiac rehabilitation improves cardiorespiratory fitness and, as a consequence, ameliorates the quality of life of patients with HF. Particularly, multiple studies demonstrated the improvement in left ventricular ejection fraction (LVEF) among patients with HF due to the various processes in the myocardium triggered by exercise. Exercise stimulates IGF-1/PI3K/Akt pathway activation involved in muscle growth in both the myocardium and skeletal muscle by regulating protein synthesis and catabolism. Also, physical activity stimulates the activation of the mitogen-activated protein kinase (MAPK) pathway which regulates cellular proliferation, differentiation and apoptosis. In addition, emerging data pointed out the anti-inflammatory effects of exercises as well. Therefore, it is of utmost importance for clinicians to accurately evaluate the patient’s condition by performing a cardiopulmonary exercise test and/or a 6-min walking test. Portable devices with the possibility to measure exercise capacity proved to be very useful in this setting as well. The aim of this review is to gather together the molecular processes triggered by the exercise and available therapies in HF settings that could ameliorate heart performance, with a special focus on strategies such as exercise-based cardiac rehabilitation.

## Introduction

Heart failure (HF) is a major cause of morbidity and mortality worldwide, with a growing prevalence mostly due to an aging population (Ziaeian and Fonarow, 2016). In fact, improvements in therapy and quality of life have increased life expectancy and, as a consequence, the percentage of the elderly in the general population, which implies an increase in age-related morbidity and comorbidity often associated with deterioration in cardiac function. The rising rate of individuals with hypertension, insulin resistance, diabetes mellitus (DM), obesity and vitamin D deficiency coupled with genetic factors, lifestyle and environmental influence pose a risk for future cardiac dysfunction and disease complications. Although it is possible to timely access asymptomatic cardiac morbidities, the diagnosis is usually delayed toward cardiac dysfunction at an advanced stage with apparent symptoms. The reason is the weak adherence of seemingly healthy individuals to regular health check-ups (Janjusevic et al., 2021).

There is strong evidence that physical exercise is beneficial for the cardiovascular system and that could prevent cardiac complications in the future (Bernardo et al., 2018). Lifestyle choices greatly affect individuals’ overall health, both those genetically pre-disposed to certain diseases and those who are not.

The aim of this review is to summarize the current understanding of the beneficial molecular processes triggered by the exercise, which lead to an improvement of the health of patients with HF and the current techniques and methods used in clinical practice that allow clinicians to accurately assess the patients’ health status. Timely assessment of the patient’s condition is the first step toward healing or amelioration of the heart function followed by therapy and tailored physical activity guided by health professionals. In addition, we have focused on the importance of vitamin D deficiency recently recognized as a risk factor for cardiovascular disease. Vitamin D has an immense role in calcium homeostasis and muscle contraction and its supplementation has been proven to lead to the improvement in muscle function, strength and athletic performance (Abrams et al., 2018).

## General Molecular Bases of Exercise

**FIGURE 1:**
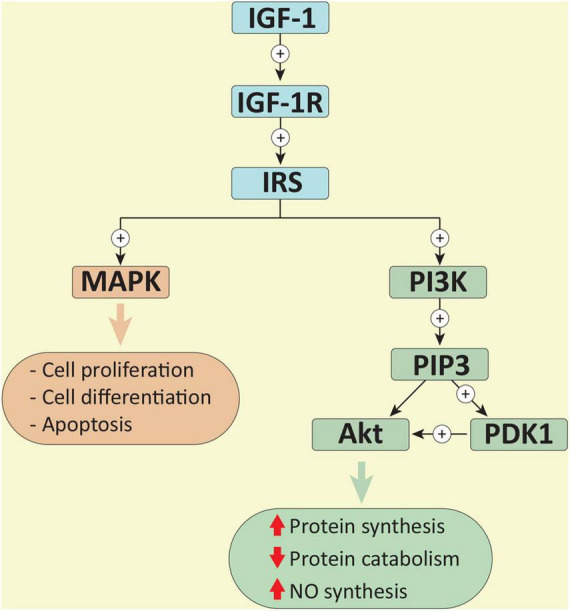
Binding to the IGF-1 receptor (IGF-1R), IGF-1 begins a series of cascading processes. Activated IGF-1R mediates the recruitment and phosphorylation of insulin receptor substrate (IRS), and the activation of the mitogen-activated protein kinase (MAPK) pathway. Phosphorylated IRS also recruits and activates phosphoinositide 3-kinase (PI3K). By phosphorylating phosphoinositide-4,5-biphosphate, PI3K creates phosphoinositide-3,4,5-triphosphate (PIP3) and consequently two docking sites for the recruiting Akt and phosphoinositide-dependent kinase 1 (PDK1). PDK1 phosphorylates Akt, leading to its activation (Schiaffino and Mammucari, 2011; Bernardo et al., 2018).

Exercise stimulates the release of growth factors such as insulin-like growth factor (IGF-1), which is responsible for the positive effects of physical activity on many cells including cardiomyocytes, endothelial cells (ECs), and immune cells (Zebrowska et al., 2009; Bernardo et al., 2018). IGF-1 binds to the IGF-1 receptor (IGF-1R) leading to its conformational change and activation by autophosphorylation and generation of a docking site for insulin receptor substrate (IRS). IRS gets to be further phosphorylated by the same receptor (Figure 1). On the one hand, phosphorylated IRS leads to the activation of the mitogen-activated protein kinase (MAPK) pathway, which is known to regulate cellular proliferation, differentiation and apoptosis (Schiaffino and Mammucari, 2011). On the other hand, phosphorylated IRS is a docking site for phosphoinositide 3-kinase (PI3K) and it is responsible for its activation as well. Activated PI3K after a series of cascading processes leads to the activation of protein kinase B (PKB, also known as Akt), the main component in this cascade (Schiaffino and Mammucari, 2011; Bernardo et al., 2018).

Besides controlling the initiation of protein synthesis, Akt regulates protein catabolism through the negative regulation of muscle ring finger 1 (MuRF1) and muscle atrophy F-box (MAFbx), which are muscle-specific components of the ubiquitin-proteasome system (Schiaffino and Mammucari, 2011; Saxton and Sabatini, 2017). While titin, nebulin, troponins, and myosin heavy chain are targeted by MuRF1, MAFbx regulates the degradation of the myogenic regulatory factor MyoD, myosin heavy chain and other sarcomeric protein (Bodine and Baehr, 2014). Taken together, the activation of the IGF-1/PI3K/Akt pathway during physical activity finely regulates both myocardial and skeletal muscle growth.

Together with exercise-induced muscle growth, communication between cardiomyocytes, ECs and vascular smooth muscle cells (VSMCs) is critical for proper adaptation to morphological and molecular changes (Bernardo et al., 2018). Following exercise-mediated myocardial hypertrophy, vascular adaptations, such as increased vessel density and changes in vascular tone, allow adequate perfusion to the heart and muscles to support the need for oxygen during physical activity (Bernardo et al., 2018). Since ECs possess IGF-1R on the cell membrane, activation of the IGF-1/PI3K/Akt pathway stimulates phosphorylation of endothelial nitric oxide synthase (eNOS) and nitric oxide (NO) production (Delafontaine et al., 2004). NO is crucial for the proper functioning of ECs and for their growth and survival, but it also has paracrine activity, which regulates the vascular tone of the VSMCs and controls oxidative stress and cardiac contraction in cardiomyocytes (Delafontaine et al., 2004; Bernardo et al., 2018).

Moreover, in the heart, there are other molecules, such as vascular endothelial growth factor (VEGF), which seem to be triggered by physical activity. Cardiomyocytes can produce VEGF which exerts its function as growth hormone in ECs, endothelial progenitor cells and mesenchymal stem cells, thus stimulating the formation of new blood vessels (Schuler et al., 2013; Bernardo et al., 2018). As IGF-1, binding to its receptor, VEGF activates the PI3K/Akt cascade, which in turn leads to NO production (Schuler et al., 2013). Furthermore, several data suggest that exercise is responsible for direct beneficial effects on vessels due to the increase in shear stress (Niebauer and Cooke, 1996). ECs possess mechanotransduction systems that enable the conversion of a mechanical stimulus into a chemical response (Chistiakov et al., 2017). The mechanosensory complex activates the PI3K/Akt signaling or activates calcium ion channels, which enable the production of NO both in peripheral vessels and in the cardiac endothelium (Schuler et al., 2013; Chistiakov et al., 2017; Bernardo et al., 2018). Among the mechanosensitive factors whose expression is modulated by shear stress, the transcription factor Klf2A deserves special mention. Indeed, this factor, which is upregulated by prolonged shear stress and -to a lower extent- by statins (van Thienen et al., 2006) exerts a protective effect on the endothelium by upregulating eNOS and NRF2 (Chistiakov et al., 2017).

It is important to emphasize that the exercise-induced signaling pathways and transcriptional responses underpinning the physiological hypertrophy are different from pathways triggered by pathological stimuli such as hypertension, valve disease or ischemic heart disease (McMullen and Jennings, 2007; Vega et al., 2017). Specifically, as mentioned above, PI3K is a mediator of physiological hypertrophy and, although in both cases the heart enlarges and wall thickens, physiological growth is different from pathological one and does not lead to severe cardiac remodeling and HF (McMullen and Jennings, 2007; Vega et al., 2017). Moreover, physiological hypertrophy is characterized by normal cardiac structure and normal or improved function (McMullen and Jennings, 2007). It is worth noting that in clinical settings it is sometimes difficult to distinguish physiological adaptations from pathological hypertrophy. For instance, due to intense training, athletic adaptations of the heart may have similar findings to pathological hypertrophy of hypertrophic cardiomyopathy, making them difficult to discriminate (Martinez and Nair, 2014).

## Effects of Exercise in Patients With Heart Failure

### The Effect of Different Types of Exercise on the Heart

Before the 1970s, physical activity was not recommended in patients with HF as symptoms of discomfort intensified after exercise (Cattadori et al., 2018). Later on, seminal studies showed that even modest physical activity has a positive impact on patients with HF (Cattadori et al., 2018). Nowadays, it is deeply ingrained that physical activity could prevent cardiac deterioration, improve the quality of life of patients with HF and be used as a predictor of survival (Pelliccia et al., 2021). It is therefore not surprising that current guidelines recommend exercise for patients with HF (U.S. Department of Health and Human Services, 2018; Pelliccia et al., 2021). The term “exercise capacity” is used to define the ability to perform exercise based on an estimate of maximal oxygen uptake (VO_2_ max or peak) (Moreira et al., 2020). VO_2_ peak is the highest amount of oxygen which can be effectively used to generate ATP by mitochondria in skeletal muscle. At the same time, VO_2_ peak is equal to cardiac output multiplied by arteriovenous oxygen content difference, as expressed by the Fick equation (Pina et al., 2003; Moreira et al., 2020). In the VO_2_ peak equation, the arteriovenous oxygen difference is mainly influenced by the endothelial and haemodynamic function and by metabolic factors, such as diabetes or cachexia (Pina et al., 2003; Aquilani et al., 2008; Khan et al., 2014). At the same time, cardiac output is expressed as heart rate (HR) multiplied by stroke volume (SV). HR trends decrease by 1 bpm per year, which partly explain the physiological decline of cardiac output and VO_2_ peak with age (Pina et al., 2003; Moreira et al., 2020). On the other hand, while SV seems to be slightly affected by age, it is consistently influenced by exercise-related morphological changes, leading to an increase in left ventricular (LV) end-diastolic volume and a decrease of LV end-systolic volume, thus promoting exercise tolerance (Pina et al., 2003; Moreira et al., 2020).

It has been reported that the increase in exercise capacity in patients with HF is a result of a combination of duration, intensity and frequency of training (Adams et al., 2017). Continuous endurance training (CET), high-intensity interval training (HIIT), and resistance training (RT) have been reported to be effective in patients with cardiac dysfunction (Adams et al., 2017). However, to date, no optimal dose has been identified for these patients (Wilson et al., 2015; Adams et al., 2017). Studies reported that RT is particularly beneficial in counteracting muscle loss in patients with HF (Adams et al., 2017), whereas CET and HIIT did not show differences in the improvement of VO_2_ and left ventricular ejection fraction (LVEF) in patients with HF with reduced ejection fraction (HFrEF) (Ellingsen et al., 2017). Given the fact that no pharmaceutical treatment has been clearly demonstrated to improve the outcomes in patients with HF with preserved ejection fraction (HFpEF), exercise-induced beneficial properties could represent a promising therapeutic approach (Adams et al., 2017). In support of these claims are results from small clinical trials pointed out that physical therapies improve exercise capacity and quality of life among patients with HFpEF (Palau et al., 2016). Interestingly, a recent study aimed to assess whether there are differences in the effects of various training including HIIT, CET, and guideline-based physical activity advice, on changes in several parameters such as VO_2_ and LVEF among patients with HFpEF, showed no significant differences (Mueller et al., 2021). Worth mentioning, effects of inspiratory muscle training were associated with improvement in exercise capacity and quality of life in patients with HFpEF (Palau et al., 2014). Therefore, further studies are needed to investigate and confirm those promising findings.

### Molecular Mechanisms Triggered by Exercise

One of the issues of this review is to disclose how the molecular processes that underlie physical activity could improve typical aspects of HF including pathological inflammation (Azevedo et al., 2016), metabolic alterations (Strom et al., 2005; Song et al., 2012), myocardial hypertrophy (Bernardo et al., 2010), fibrosis (Wilson et al., 2015; Nystoriak and Bhatnagar, 2018), and oxidative stress (Azevedo et al., 2016).

In healthy individuals, the “fight or flight” response is mediated by catecholamines (i.e., adrenaline and noradrenaline), which are produced by the medulla of the adrenal gland after stimulation by motor centers in the brain (Adameova et al., 2009). In patients with HF, the renin-angiotensin-aldosterone system (RAAS) is responsible for over-activation of the sympathetic nervous system and excessive production of catecholamines, causing damage to the cardiovascular system (Borovac et al., 2020). Decreased concentration of catecholamines, angiotensin II (Ang II) and aldosterone in patients with HF due to the training have been encouraging for the control of inflammation and myocardial damage (Coats et al., 1992; Braith et al., 1999; Gielen et al., 2010). Furthermore, impulses from working muscle and motor centers in the brain stimulate the hypothalamus-pituitary-adrenal axis, which mediates the production of the adrenocorticotropic hormone followed by the release of cortisol from the cortex of the adrenal glands (Gleeson et al., 2011; Cruz-Topete and Cidlowski, 2015). Following binding to the glucocorticoid receptor (GR), cortisol controls gene expression by direct binding to specific sequences of DNA or by interaction with other transcription factors (Cruz-Topete and Cidlowski, 2015). Also, GR signaling has been reported to inhibit pro-inflammatory cytokines expression by tethering itself to the nuclear factor kappa light chain enhancer of activated B cells (NF-κB) (Cruz-Topete and Cidlowski, 2015).

In the same context, depending on the duration and intensity of the exercise, it is assumed that other mechanisms cause anti-inflammatory effects (Gleeson et al., 2011). Since exercise is reported to control visceral fat, it could counteract the systemic inflammation by reducing pro-inflammatory cytokines such as tumor necrosis factor-alpha (TNF-α), interleukin (IL)-1β, and IL-6 (Gleeson et al., 2011). Furthermore, during and following exercise, skeletal muscle releases IL-6 whose concentration returns to resting level after physical activity (Pedersen, 2009; Gleeson et al., 2011). IL-6 acts as both pro-inflammatory and anti-inflammatory cytokine; in this last case, it stimulates the release of cortisol and cytokines such as IL-10 and IL-1 receptor antagonist (IL-1RA) (Pedersen, 2009; Gleeson et al., 2011; Scheller et al., 2011). Last, as anticipated, laminar blood flow induces the expression of Klf2, a transcription factor that maintains EC homeostasis, quiescence and exerts anti-thrombotic and anti-inflammatory activities (Chistiakov et al., 2017).

In addition, the electron transfer activity, which leads to ATP production, is partly responsible for the production of reactive oxygen species (ROS) (Bernardo et al., 2018). Under physiological conditions, ROS act as signaling molecules and their over-production are compensated by antioxidant systems such as superoxide dismutase (Haram et al., 2008). Oxidative stress is defined as the increase in ROS due to the imbalance between their production and neutralization (Bernardo et al., 2018). NADPH oxidase, uncoupled NOS activity and oxidative reaction in mitochondria are the main source of ROS (Bernardo et al., 2018). In numerous metabolic diseases and HF, excess ROS leads to cell death, inflammation, fibrosis and mitochondrial dysfunction (Kojda and Hambrecht, 2005; Azevedo et al., 2016) due to the persistently oxidative stress that is not properly quenched (Haram et al., 2008). Conversely, exercise appears to cause a pulsatile increase in ROS and a transient increase in oxidative stress, which might have a therapeutic effect in patients with HF (Bernardo et al., 2018). Finally, Klf2, induced by moderate exercise training, drives the expression of the antioxidant transcription factor nuclear factor erythroid 2-related factor 2 (Nrf2) in vascular ECs, that, in turns, upregulates several antioxidant genes including heme oxygenase-1, sequestosome 1, NAD (P)H quinone oxidoreductase 1, ferritin (heavy and light chains), microsomal epoxide hydrolase, glutathione S-transferase, thioredoxin 1, and c-glutamylcysteine synthase (Chistiakov et al., 2017; Sangha et al., 2021). However, observations of individuals engaged in high-intense, long-term exercise suggest that these types of physical activity may have a negative impact on heart physiology. Indeed, atrial fibrillation, coronary calcification and cardiac fibrosis are increased in older athletes with respect to their inactive counterparts (Eijsvogels et al., 2016). Although additional research is needed for the exact mechanism, it is likely that in some individuals negative adaptations could be explained by induction of inflammation, endothelial dysfunction, and atherosclerosis due to signals induced by prolonged resistance exercises (Eijsvogels et al., 2016).

**FIGURE 2:**
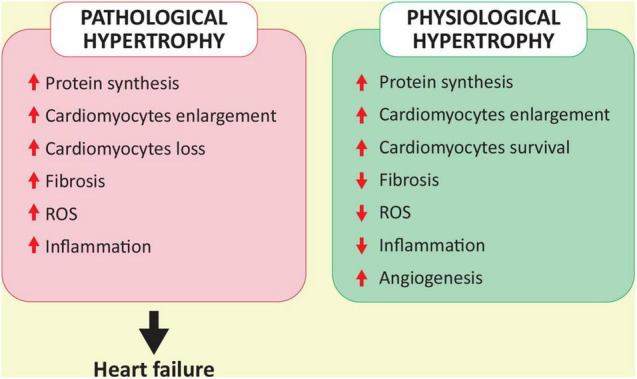
A schematic representation of the comparison between pathological and physiological hypertrophy.

Furthermore, exercise has an important role in the regulation of cardiac metabolism, allowing adaptation to changes of systemic demand. Particularly, cardiac metabolism is mainly based on the consumption of fatty acid, followed by glucose, lactate and other substrates such as ketone bodies, pyruvate, acetate, and branched-chain amino acids (Fulghum and Hill, 2018). The heart has a high demand for ATP and the amount and type of substrate used by the heart change in response to energy demand (Fulghum and Hill, 2018). The number of mitochondria in combination with the balance of glycolysis, glucose and fatty acid oxidation is fundamental for the proper production of ATP (Bernardo et al., 2018). Hypertension, DM and myocardial infarction are characterized by the uncoupling of glucose utilization and glucose oxidation (Strom et al., 2005). Altered metabolism combined with impaired angiogenesis, cardiomyocyte death and overexpression of cytoskeletal and extracellular matrix genes leads to fibrosis and pathological hypertrophy (Wilson et al., 2015; Nystoriak and Bhatnagar, 2018). On the other hand, in active individuals, especially athletes, the combination of cardiomyocyte enlargement, extracellular matrix regulation and vascular and mitochondrial adaptation to exercise can lead to physiological hypertrophy (Bernardo et al., 2018). Conversely to diseased conditions, the expression of genes of glucose/insulin pathway, lipid metabolism and cell survival in trained rodents are not impaired due to the physiological hypertrophy (Bernardo et al., 2018). Therefore, exercise leads to beneficial cardiac hypertrophy that is not associated with cardiac dysfunction as in pathological hypertrophy (Figure 2). Since the diseased heart has impaired metabolism (Bernardo et al., 2018), the resulting inadequate ATP synthesis could be at least partially recovered with exercise. Indeed, during exercise, lipolysis in adipose tissue mediates an increase in circulating free fatty acid, thus providing the heart with the primarily substrate (Fulghum and Hill, 2018; Moreira et al., 2020). On the other hand, glucose concentration tends to increase depending on the type and duration of physical activity (Fulghum and Hill, 2018). In the heart, changes in gene expression promote both the uptake and use of free fatty acid and glucose as well as mitochondrial homeostasis (Bernardo et al., 2018; Fulghum and Hill, 2018; Moreira et al., 2020). Additionally, the increase in circulating catecholamines due to physical activity contributes to the oxidation of stored glucose and lipid metabolism (Fulghum and Hill, 2018; Moreira et al., 2020).

### Cumulative Effects of Exercise on Left Ventricular Ejection Fraction, Fibrosis, and Cardiomyocyte Proliferation

Lastly, it is important to mention that numerous studies have reported that exercise has a positive impact on LVEF among patients with HF (Adams et al., 2017). The positive outcome of the exercise in counteracting myocardial remodeling could be the sum of the effects on vessels and direct molecular changes in the myocardium (Adams et al., 2017). Based on the aforementioned effect of exercise on signaling pathways, it is plausible that training could regulate cell growth by supporting protein synthesis and downregulating their catabolism through inhibition of the E3 ubiquitin ligases MuRF1 and MAFbx (Schiaffino and Mammucari, 2011; Bodine and Baehr, 2014). The decrease in Ang II and aldosterone concentration in patients with HF after training offers a promising scenario in reducing fibrosis in diseased hearts (Coats et al., 1992; Braith et al., 1999; Gielen et al., 2010). Moreover, in patients with HF, physical activity could have positive effects on health by opposing skeletal muscle atrophy, which is often experienced by patients with HF due to prolonged immobilization. Furthermore, studies on animal models sustain that exercise could also induce the proliferation of cardiomyocytes (Bernardo et al., 2018). Given the limited regenerative capacity of the heart in mammalians, this result seems very attractive because the same mechanism could be inferred even in humans, albeit it is still debated.

Despite the aforementioned positive properties of the training programs, adherence to this therapeutic practice is still limited due to other factors such as age, comorbidities, psychologic, and socio-economic conditions (Cattadori et al., 2018). In some cases, the pharmacological approach seems more easily accepted by patients with HF or the only possible option. The use of a single pill that has the same positive effect as exercise is desirable, but difficult to achieve (Bernardo et al., 2018). Vitamin D supplementation could be evaluated to influence downstream effectors of the IGF-1/PI3K/Akt pathway. However, it should be considered that constitutive activation of the pathway has been reported as a cause of pathological hypertrophy, fibrosis, cardiac dysfunction and tumor development (Bernardo et al., 2018). It is now evident that the pulsatile activation of the IGF-1/PI3K/Akt pathway is responsible for the beneficial effects of training (Bernardo et al., 2018).

## Vitamin D Deficiency

### Multiple Roles of Vitamin D and Their Association With Cardiovascular Diseases and Diabetes Mellitus

Vitamin D deficiency has been demonstrated to be associated with the risk of development and the severity of cardiovascular diseases and DM, which are both conditions characterized by intolerance to exercise and muscle fragility (Witham, 2011; Lopes et al., 2014; Aleksova et al., 2020). The prevalence of vitamin D deficiency numbers more than a billion people worldwide (Sirajudeen et al., 2019). Recent studies have shown that vitamin D plays a role in muscle function and strength, as well as that higher levels of vitamin D decrease injury rate and enhance athletic performance (Abrams et al., 2018). In a study by Lopes et al. (2014) the results pointed out that patients diagnosed with HF, DM, and vitamin D insufficiency have lower physical function and strength than patients without DM and optimal vitamin D concentration.

Vitamin D is a steroid hormone that is mostly endogenously produced in the skin after exposure to UV radiation (90%), while the rest comes from food (Mendes et al., 2018). Besides being formerly known for its important role in regulating calcium absorption and bone mineralization (Holick, 2007), thanks to extensive research, it is now known that vitamin D plays important roles in other processes, such as the regulation of gene expression of proteins involved in cell proliferation, differentiation, DNA repair and apoptosis (Holick, 2007; Graziano et al., 2016). Along with genomic actions, vitamin D performs non-genomic ones involving stimulation of transmembrane second messenger systems, such as Akt, protein kinase A (PKA), protein kinase C (PKC), proto-oncogene tyrosine-protein kinase Src, and MAPK (Boland, 2011; Hii and Ferrante, 2016). The vitamin D receptor, through which vitamin D performs its functions, has been found in various tissues and organs such as cells of the immune system, gastrointestinal tract, liver, pancreas, heart (Holick, 2007), and skeletal muscle (Abrams et al., 2018), which explain the vitamin D involvement in immune response modulation (Guillot et al., 2010), its association with glucose homeostasis, DM (Yaribeygi et al., 2020), obesity, hypertension, and cardiovascular disease (Sirajudeen et al., 2019).

In muscle cells, vitamin D is crucial for calcium homeostasis and muscle contraction, as well as for skeletal muscle growth. Biopsies on vitamin D deficient patients showed the atrophy of type II muscle fibers (Ceglia, 2009). Not surprisingly, myopathy has been noted among patients with vitamin D deficiency (Boland, 2011). Also, patients with HF, DM, and vitamin D deficiency are strongly associated with muscle impairment (Lopes et al., 2014). Furthermore, a study with 572 vitamin D deficient patients confirmed the correlation between low vitamin D levels and muscular pain, headache, and fatigue (Knutsen et al., 2010). Conversely, vitamin D supplementation improves musculoskeletal performance in people with vitamin D deficiency. Specifically, studies conducted in elderly vitamin D deficient individuals have shown that vitamin D supplementation leads to an increase in muscle strength and physical function (Cannell et al., 2009). Moreover, several studies demonstrated the beneficial effects of vitamin D supplementations in terms of prevention in the development of cardiac disease and unfavorable outcomes (Mirhosseini et al., 2018; Acharya et al., 2021; Feuchtner et al., 2021). However, it is important to mention the inconsistency regarding the results obtained by studies that aimed to assess the positive effects of vitamin D supplementation. Even though studies indicated a strong association between vitamin D deficiency and the risk of developing cardiovascular disease (Judd and Tangpricha, 2009) and type 2 DM (Park et al., 2018; Mohammadi et al., 2021), as well as with poor outcomes associated with the diseases (Dai et al., 2021), studies involving vitamin D supplementation did not always yield results in favor of significant post-supplementation improvement among participants (Scragg et al., 2017; Zittermann et al., 2017; Manson et al., 2019). A recent meta-analysis involving more than 83,000 individuals did not show an association between vitamin D supplementation and a reduced risk of cardiovascular events and mortality (Barbarawi et al., 2019). The possible explanations underlying this controversial data could be due to the limitations of the study design such as inadequate or infrequent dosage or short duration. In the same context, it is worth mentioning that in a study conducted by Boxer et al. (2013) vitamin D supplements over a period of 6 months did not improve exercise capacity according to VO_2_ peak, 6-min walking test (6MWT), and isokinetic muscles strength despite a strong increase in serum vitamin D. However, the limitations of this study were short duration and small cohort, therefore further clinical trials with large sample size and longer follow-up of participants might yield different results. By all means, future studies are needed to respond to those questions.

### Vitamin D Deficiency and Its Effects on the Heart

The mechanisms by which vitamin D deficiency affects the pathophysiology of HF are multiple and overlap. Vitamin D reduces the level of pro-inflammatory cytokines such as IL-1β, IL-6, and TNF-α by inhibiting NF-κB activity (Kassi et al., 2013). Therefore, by promoting the anti-inflammatory state, vitamin D prevents atherosclerosis development (Spagnoli et al., 2007). Accumulating evidence proved that vitamin D regulates RAAS (Ajabshir et al., 2014) by suppressing both *renin* and *angiotensinogen* genes using different mechanisms (Kassi et al., 2013). By vitamin D–responsive element in the promoter of the *renin* gene, vitamin D suppress renin expression whereas by blocking the N F-κB pathway, down-regulates the expression of the *angiotensinogen* gene. This finding is extremely important since RAAS regulates blood pressure maintaining normal cardiovascular functions and is directly involved in the development of HF (Kassi et al., 2013). Increased concentrations of renin and Ang II have been found among individuals with vitamin D deficiency (Santoro et al., 2015).

Furthermore, it has been noted that vitamin D is involved in the regulation of glucose homeostasis by stimulating insulin synthesis and its secretion and that in cases of vitamin D deficiency the insulin secretion is altered (Palomer et al., 2008). This finding is extremely important taking into consideration that vitamin D deficiency is very common among patients with DM (Aleksova et al., 2020), who have a higher tendency of developing coronary artery disease and myocardial infarction. Indeed, the elevated glucose concentration causes low-grade inflammation due to oxidative stress, leading to endothelial dysfunction and resulting in atherogenesis (De Rosa et al., 2018). Moreover, our group recently showed that among patients suffering from myocardial infarction, vitamin D and DM have important prognostic value, individually and synergistically, in terms of poor outcomes (Aleksova et al., 2020). Therefore, is not surprising that vitamin D deficiency is associated with disease complication among patients with DM. Also, hyperglycemia stimulates RAAS and Ang II-induced expression of pro-inflammatory cytokines, which are expressed as well *via* the N F-κB pathway due to the deficiency of vitamin D (Aleksova et al., 2020). Since in normal conditions these aspects would be suppressed, the cumulative beneficial effects of vitamin D contribute to overall health. Intriguingly, experimental studies, conducted on streptozotocin-induced diabetes in rats, showed that swimming was associated with an increase in vitamin D serum levels and increased vitamin D receptors in the pancreas, skeletal muscle and adipose tissue (Aly et al., 2016).

**FIGURE 3:**
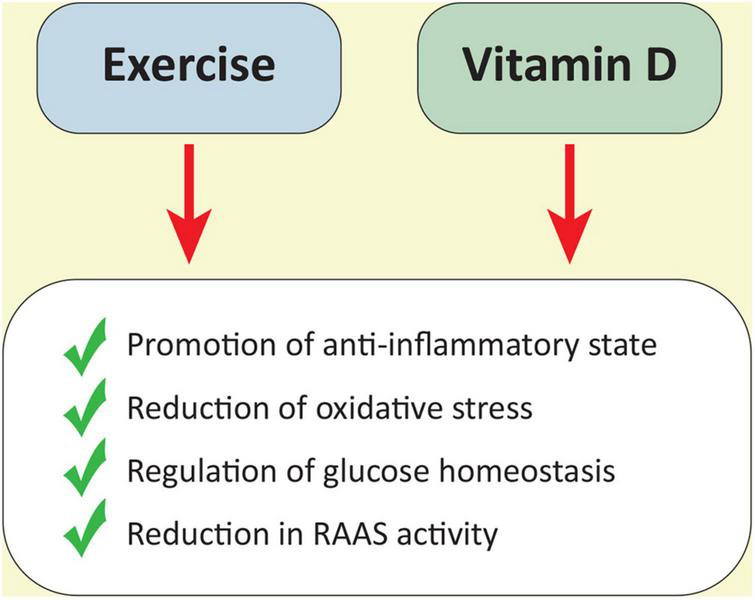
A schematic representation of beneficial processes triggered by both exercise and the vitamin D activity.

Given that most vitamin D is produced after exposure to UV radiation and that only 30 min of sun exposure twice a week are necessary for sufficient synthesis (Aleksova et al., 2015), outdoor exercise may guarantee all the aforementioned favorable effects of physical activity combined with higher vitamin D endogenous synthesis. The beneficial processes that are common to physical exercise and vitamin D activity are represented in the Figure 3.

## Current Available Exercise Testing

In an era of advanced diagnostic techniques and complex prognostic models, exercise testing remains a key investigation in Cardiology due to its cost-effectiveness, non-invasiveness and potential applicability in multiple settings of cardiovascular medicine. Exercise stress tests are indeed recommended as part of routine screening in competitive athletes, as well as for functional evaluation and risk stratification in individuals with cardiomyopathies and chronic HF. Furthermore, systematic physical activity should be considered as a full-fledged therapeutic strategy, capable of improving overall cardiometabolic health (U.S. Department of Health and Human Services, 2018) and representing the pivot around which the cardiac rehabilitation rotates after acute coronary syndromes and cardiac surgery (Anderson et al., 2016).

The 6MWT represents the simplest form of exercise testing, and it is widely used in different areas of clinical practice (Table 1). It measures the distance covered over a time of 6 min, walking as fast as possible on a flat surface, under the supervision of a doctor or a physiologist (Enright, 2003). Several studies have been made to determine the normal reference values on 6MWT, and the predicted 6MWD (The 6-min walking distance) can now be estimated using mathematical equations which accounts for several factors such as age and sex (Enright and Sherrill, 1998). The 6MWT is particularly useful when other exercise tests are not feasible, for example in elderly subjects or in patients with advanced cardiac disease. In the context of pulmonary hypertension, the 6MWD is also listed among the variables used to stratify patients’ risk and to direct medical therapy (Boucly et al., 2017). The data of the total distance walked during the test are supplemented by the evaluation of exercise-induced changes in blood pressure, HR and peripheral arterial oxygen saturation *via* pulse oximetry. The modified 0–10 Borg Scale (MBS) is commonly used to quantify the subjective degree of dyspnea and fatigue in patients undergoing 6MWT. In summary, the 6MWT is a reliable and convenient tool that provides quick information on the aerobic capacity and endurance of patients with cardiopulmonary conditions. Currently, new mobile phone-based systems are emerging with the aim to allow patients to perform the 6MWT on their own, reducing the exploitation of hospital staff and facilities (Salvi et al., 2020). This is of extreme importance nowadays, in the COVID-19 era, which is characterized by a strong limitation of regular hospital visits.

**TABLE 1:** Summary of performances and benefits of different tests to assess patient health.

Test	Performance	Advantages and usefulness	References
6-min walking test (6MWT)	Distance measurement over 6 min	• Simple and easy to perform, especially when other tests are not feasible • Quick and convenient	Enright, 2003; Boucly et al., 2017
Handgrip strength (HGS)	Maximum isometric force generated by the muscles of the hands and forearm	• To assess the progress of rehabilitation during follow-up • Prevention • Fast and cheap	Leong et al., 2015
Cycle ergometry and treadmill testing	12-lead ECG recording + blood pressure + symptoms monitoring	• To prescribe physical activity • Standardized protocols to detect myocardial ischemia, inducible arrhythmias, and abnormal pressure responses during effort	Vilcant and Zeltser, 2021
Cardiopulmonary exercise testing (CPET)	Exercise testing (involving cardiovascular, skeletal muscle and metabolic responses) + ventilation and gas exchange information (respiratory response)	• To establish the cause of unexplained dyspnea and/or exercise intolerance • To improve diagnostic accuracy for detecting inducible myocardial ischemia, peripheral vascular disease, arterial hypoxaemia • For identification of high-risk patients who are candidates to cardiac transplantation or VAD implantation • Predict outcome in patients with heart failure • To investigate the effects of therapeutic interventions on functional capacity • To optimize prescription of exercise training • Evaluation of the integrative exercise responses comprising various systems (cardiovascular, pulmonary, skeletal muscle etc.)	Belardinelli et al., 2003; Mezzani, 2017; Laveneziana et al., 2021; McDonagh et al., 2021

Handgrip Strength (HGS) is another basic functional test used in a range of clinical settings. It measures the maximum isometric force generated by hands and forearm muscles in a fast and cheap way. In an analysis from the Prospective Urban-Rural Epidemiology (PURE) study, low grip strength was found to be significantly associated with cardiovascular mortality (Leong et al., 2015), suggesting the use of the HGS test in the context of rehabilitation and prevention.

More comprehensive methods of cardiovascular stress testing are represented by cycle ergometry and treadmill testing, which provide additional information due to the 12-lead electrocardiogram (ECG) recording along with blood pressure and symptoms monitoring. These forms of stress testing are generally performed according to standardized protocols, the most notable of which is the Bruce protocol. Besides the diagnostic and prognostic value, the American College of Cardiology/American Heart Association (ACC/AHA) states that exercise stress testing can be useful for activity prescription (Vilcant and Zeltser, 2021), especially with respect to the estimated exercise capacity, which is expressed in metabolic equivalents (METs) and is the only treadmill variable associated with all-cause mortality (Goraya et al., 2000).

Cardiopulmonary exercise testing (CPET) integrates the data derived from conventional exercise testing with ventilation and gas exchange information, thus allowing a comprehensive evaluation of both the uptake, transport and use of oxygen during exercise (Mezzani, 2017). Among the most widely used parameters derived from cardiopulmonary exercise are VO_2_ peak, VCO_2_ to VO_2_ ratio and Ventilation (VE)/VCO_2_ slope. The VCO_2_ to VO_2_ ratio, also called Respiratory Exchange Ratio, is a valuable and objective tool for defining whether the test was maximal or not. In fact, a VCO_2_ to VO_2_ greater than 1 reflects the switch from an aerobic to anaerobic metabolism with the production of lactic acid. As mentioned above, VO_2_ peak is obtained by multiplying the cardiac output and the arteriovenous difference in oxygen content at the exercise peak and it has been demonstrated to be a better descriptor of exercise tolerance as compared to other parameters derived by conventional exercise testing. However, the VO_2_ peak lacks specificity, because all the diseases affecting oxygen transport and/or oxygen use during exercise could produce a reduction in the predicted VO_2_ peak. Therefore, a decreased predicted VO_2_ peak is commonly found in patients with HF as well as in those with pulmonary diseases, anemia, or more rarely mitochondrial affections, but it can also reflect a state of deconditioning (Mezzani, 2017). Finally, the VE/VCO_2_ slope is a useful parameter to evaluate patient-ventilator efficiency because it describes the amount of air a patient must ventilate to eliminate one liter of CO_2_. The slope is usually highly displaced in patients with HF as well in those with pulmonary hypertension, and a correlation between progressively higher values and increasing disease severity have been found (Mezzani, 2017).

In the field of HFrEF, CPET has been traditionally used to identify patients at high risk who are candidates to cardiac transplantation. However, over the years, scientific evidence has demonstrated the utility of cardiopulmonary testing in several other contexts of HF such as HFpEF or to address more compromised patients to other advanced treatments such as LV assist devices (Laveneziana et al., 2021; McDonagh et al., 2021). Notably, the possibility of repeating this test over the time allows clinicians to achieve a good assessment of treatment effectiveness, thereby facilitating the management of HF (Corra et al., 2018). Furthermore, there are growing data demonstrating the prognostic power of cardiopulmonary testing in the specific setting of HF patients contextualized for the etiology of cardiomyopathy (Sinagra et al., 2020). In patients with Dilated Cardiomyopathy, parameters derived from CPET such as VE/VCO_2_ slope and peak VO_2_% (the percentage of VO_2_ obtained compared to the theoretical VO_2_ max for age, weight, sex, and ethnicity) have recently emerged as predictors of cardiovascular death/heart transplantation (Sinagra et al., 2016). CPET has recently demonstrated to be safe also in patients with arrhythmogenic cardiomyopathy with VE/VCO_2_ slope correlating with a worse outcome in these patients (Scheel et al., 2020).

Lastly, compared with traditional ECG stress testing, CPET improves diagnostic accuracy for identifying exercise-induced myocardial ischemia in patients with coronary artery disease. Indeed, gas exchange analysis can be used to detect a cardiac output depression caused by myocardial ischemia during exercise, especially when the ECG is uninterpretable (Belardinelli et al., 2003).

Nevertheless, although providing additional impactful data in comparison with standard stress tests, CPET is characterized by greater technical complexity, since it requires specific equipment including oxygen and carbon dioxide gas analysers, as well as dedicated and intense training for medical officers. Additionally, compared with traditional exercise tests, CPET is more expensive and time-consuming (Wong et al., 2018).

Despite the need for further studies to explore any potential application of CPET, this exam appears to be a promising tool in the risk stratification of patients with several different cardiovascular diseases. It must be acknowledged that all the aforementioned parameters derived from the different exercise testing modalities are subjected to several possible errors, so there is a need to always interpret these measurements in light of the clinical context.

Exercise stress tests can also be used to prescribe the intensity of exercise for patients enrolled in cardiac rehabilitation programs, which are strongly recommended by the latest European guidelines for various cardiovascular conditions (Pelliccia et al., 2021). Specifically, ventilatory thresholds derived from CPET allow to point out exercise intensity with a highly personalized approach (Hansen et al., 2019). In theory, all patients who have experienced an acute coronary syndrome, cardiac surgery, or percutaneous intervention could benefit from an early exercise-based rehabilitation program to reduce the risk of cardiovascular death and rehospitalization. Tailored exercise training programs are also suggested in all stable patients with chronic HF, including those with preserved LVEF, and in all individuals undergoing heart transplantation or ventricular assist device (VAD) implantation. Regular physical activity represents a cornerstone in the holistic prevention of atrial fibrillation, as it is an integral part of the therapeutic management of the atherosclerotic peripheral arterial disease. Lastly, the beneficial action of constant moderate exercising also in primary cardiovascular prevention must never be forgotten.

## Conclusion

There is a growing body of evidence supporting the beneficial effects of exercise on the heart muscle in both healthy individuals as well as patients with cardiovascular disease. Several studies pointed out the improvement of LVEF among patients with HF due to exercise-induced myocardial remodeling. Exercise leads to physiological hypertrophy through the growth and strengthening of cardiomyocytes along with increased vessel density and changes in vascular tone enabling adequate perfusion of the heart to support the need for oxygen. Exercise triggers different pathways in the myocardium and skeletal muscle regulating both protein synthesis and catabolism. Moreover, recent studies in animal models have suggested that exercise could induce cardiomyocyte proliferation, which is still debatable. However, what we can conclude with certainty is that supervision tailored physical activity have a positive impact on the heart of the patients with HF. Worth mentioning, vitamin D is important for calcium homeostasis and muscle contraction and its supplementation could improve muscle strength and physical function. In addition, vitamin D has been recognized as an emerging prognostic biomarker in terms of adverse outcomes among patients with myocardial infarction. More, it has been shown that endurance exercise could increase the circulating levels of 25-hydroxyvitamin D (Sun et al., 2017) and may increase vitamin D signaling *via* its receptor as well (Lithgow et al., 2021). Last, due to the fact that 90% of vitamin D is endogenously produced in the skin after exposure to UV light, appropriate aerobic physical activity outdoors would give the best results.

In addition, vitamin D, as well as physical activity, reduce the concertation of pro-inflammatory cytokines. Therefore, choosing a healthy lifestyle in terms of regular moderate, physical activity should be an integral part of our routine for preventing heart dysfunction in combination with vitamin D supplementation, if necessary.

## Author Contributions

AA, MJ, and ALF contributed to the conception of the manuscript. AA, MJ, GG, AP, and ALF wrote and prepared the original draft. AA, MJ, GG, AP, LP, CC, ALF, APB, and GS reviewed and edited the manuscript. MJ and ALF created the images. All authors have read and agreed to the published version of the manuscript.

## Conflict of Interest

The authors declare that the research was conducted in the absence of any commercial or financial relationships that could be construed as a potential conflict of interest.

## Publisher’s Note

All claims expressed in this article are solely those of the authors and do not necessarily represent those of their affiliated organizations, or those of the publisher, the editors and the reviewers. Any product that may be evaluated in this article, or claim that may be made by its manufacturer, is not guaranteed or endorsed by the publisher.
